# Supercritical Techniques for Pharmaceutical Applications

**DOI:** 10.3390/pharmaceutics16121551

**Published:** 2024-12-04

**Authors:** Marta Gallo, Barbara Onida, Mauro Banchero

**Affiliations:** Department of Applied Science and Technology, Politecnico di Torino, Corso Duca degli Abruzzi 24, 10129 Torino, Italy; marta.gallo@polito.it (M.G.); barbara.onida@polito.it (B.O.)

## 1. Introduction

Supercritical fluid technology is an innovative approach that has been extensively explored in various research fields, since it offers a way to limit or replace the use of organic solvents in numerous industrial processes [1]. This is particularly appealing to the pharmaceutical sector, where the removal of harmful chemicals from drug formulations is fundamental for promoting sustainability and enhancing product quality [2]. Among the available alternatives, supercritical carbon dioxide (scCO_2_) is the most widely used since it is economical, non-toxic, recyclable and non-flammable. Its mild critical conditions allow thermolabile pharmaceutical compounds to be easily processed [2,3]. The combination of its gas-like properties (i.e., high diffusivity and low viscosity) and liquid-like characteristics (i.e., solvent power), which can also be tuned by simply varying temperature and pressure, has led to the development of a wide range of techniques for pharmaceutical applications [4], as shown in the papers published in this Special Issue.

## 2. Overview of the Published Contributions

One of the most remarkable uses of supercritical fluids is the production of aerogels. Gels dried under supercritical conditions result in light materials with unique properties, such as high surface area and uniform pore size distribution. Regarding pharmaceutical applications, aerogel carriers can enhance drug stability and improve the drug dissolution rate or its selectivity for target tissues [5]. In this context, aerogels derived from natural polysaccharides represent a promising alternative for drug delivery, particularly for colonic administration, as clearly highlighted in the review by Illanes-Bordomás and coworkers (contribution 1). In fact, the aerogel characteristics are combined with the physicochemical properties of polysaccharides, which impart the enzymatic and/or pH resistance necessary for colonic drug delivery. The obtained carriers, therefore, are effective systems able to load, protect, and release drugs in a controlled manner. In their work, the authors provide an overview of the different methods used to prepare and drug-load polysaccharide-based aerogels. They point out that developing coated aerogels is the most successful strategy for targeting the colon while minimizing premature drug release. Techniques such as fluidized-bed or spouted-bed coaters, alongside the use of coaxial nozzles and supercritical drying, have provided the best coating results. However, challenges remain in achieving the incorporation of poorly soluble drugs or other New Molecular Entities at appropriate doses. Furthermore, the crystalline state of the loaded drug and its changes during storage are still unclear, and the mucoadhesive properties of the aerogels require further investigation. According to the authors, future research should focus on conducting in vivo studies and exploring the impacts of microbiota on the drug release process (contribution 1).

The poor water solubility of many active ingredients is a major challenge for pharmaceutical companies, since it limits drug bioavailability. Among the various strategies employed to address this issue, the use of supercritical technology to achieve drug micronization is a promising alternative, as it can be also combined with the incorporation of the drug into appropriate carriers [2,6]. The available supercritical processes can be categorized based on the different roles of scCO_2_: it can act as a solvent in processes such as the Rapid Expansion of Supercritical Solutions (RESS) or the Supercritical Fluid Extraction of Emulsions (SFEE), as an antisolvent in the Supercritical Antisolvent (SAS) approach, or as a co-solute using techniques like Supercritical-Assisted Atomization (SAA) [2,4,7]. These three strategies are well represented by the other papers featured in this Special Issue.

In the RESS process, the active principle is first dissolved in scCO_2_, which is then depressurized through a nozzle. This leads to a density drop, resulting in high supersaturation and subsequent particle precipitation [2]. Using this technique, Sharmat and coworkers (contribution 2) synthesized a novel, amber-colored, viscous aqueous solution of the anticancer drug cisplatin. The authors refer to the resulting product as “liquid” cisplatin, which consists of a highly solvated network of stable cisplatin nanoclusters in water, exhibiting 27 times greater water solubility than the standard drug. Moreover, “liquid” cisplatin was found to be stable at ambient conditions for over a year. Cell viability and apoptosis studies conducted on human lung adenocarcinoma A549 cells showed the potential of this formulation to achieve a more sustained anticancer effect with respect to standard cisplatin.

SFEE is a technique where scCO_2_ acts as an extracting solvent. First, a water-in-oil-in-water (W/O/W) emulsion containing all pharmaceutical compounds is prepared. This W/O/W emulsion is then brought into contact with scCO_2_, which extracts the organic solvent, leading to the formation of the final particle suspension [7]. In his paper (contribution 3), Park investigates the correlation between the process variables of this complex technique and the main characteristics of the obtained particles. This correlation is essential to guarantee good control of drug release profiles. To pursue this goal, bovine serum albumin (BSA) was selected as a test molecule for encapsulation in poly(lactic-co-glycolic acid) (PLGA) microspheres via SFEE technology. The examined process variables included those related to emulsion preparation (e.g., homogenization speed and emulsification time), as well as those connected to the supercritical extraction step (e.g., temperature, pressure, etc.). The author found that the variations in these parameters could influence the particle size and morphology, as well as the encapsulation efficiency (EE) and initial drug burst release (IBR) of BSA. On the whole, it was observed that when the EE of the prepared microspheres was low, a higher proportion of BSA was located on their external surface, leading to a larger IBR (contribution 3).

The SAS approach, in which scCO_2_ acts as an antisolvent, is probably the most widely used technique in the literature for obtaining particle precipitation. This is evidenced by the fact that three out of the seven contributions to this Special Issue employ this technique (contribution 4, contribution 5, contribution 6). SAS is based on the poor solubility of an active compound in scCO_2_. The process involves dissolving the active compound (often in combination with a carrier) in an organic solvent that is readily miscible with scCO_2_. When the solution is in contact with the scCO_2_, the supercritical medium dissolves into the organic solvent, thereby reducing its solvent power towards the pharmaceutical solutes, which ultimately precipitate [2,6]. Ha and coworkers (contribution 4) focused their research on the preparation of nanoparticles of the pure drug telmisartan (a medication used to treat hypertension) without the presence of any carrier. Unlike most studies that have made use of the SAS process, the drug was dissolved in a solvent mixture (dichloromethane and methanol) rather than a single organic solvent before being contacted with scCO_2_. The use of mixed solvents can enhance drug dissolution during the initial phase of the process, which facilitates the subsequent antisolvent precipitation of nano- and microparticles. Furthermore, the morphologies and sizes of the particles can be easily controlled by adjusting the composition of the solvent mixture. The authors employed fractional factorial design to investigate the influence of SAS process parameters on the size and morphology of the obtained particles. They found that reducing particle size and achieving an amorphous state for the precipitated drug could enhance its dissolution rate, resulting in higher in vivo oral bioavailability in rats compared to unprocessed telmisartan. García-Sobrino and coworkers (contribution 5), instead, employed the SAS technique to incorporate icariin, a molecule that stimulates the differentiation process in osteoblastic cultures, into N-vinyl caprolactam (VCL) carrier nanoparticles. These nanoparticles were then used to dope thermosensitive hydrogels to be employed as bone-cell-harvesting platforms. Their study identified optimal formulations for icariin-activated hydrogels, and successful transplantation and recultivation of osteoblastic sheets were achieved. On the other hand, Mottola and De Marco (contribution 6) compared the performances of two different carriers, polyvinylpyrrolidone (PVP) and β-cyclodextrin (β-CD), in the preparation of SAS-processed powders containing curcumin, a natural active principle with poor water solubility. While PVP is one of the biocompatible polymers most commonly used as a drug carrier, β-CD is a cyclic oligosaccharide with a hydrophilic truncated cone-shaped exterior and a hydrophobic conical cavity where the drug can be encapsulated. In the study, both carriers significantly accelerated the dissolution rate of curcumin compared to the unprocessed active principle. Notably, the use of β-CD proved to be more advantageous, as it ensured rapid release with a lower amount of carrier in the formulation.

The work by Mottola and De Marco (contribution 6) pointed out growing interest in the literature [8] in achieving the complexation of drugs with cyclodextrin carriers through a green supercritical medium rather than conventional techniques. This trend is further supported by the last paper of this Special Issue (contribution 7), in which Wu and coworkers investigated the encapsulation of Beclomethasone dipropionate, a glucocorticosteroid drug for respiratory diseases, into γ-cyclodextrin (γ-CD). In the study, however, the drug/cyclodextrin complexation was obtained through a different technique, SAA, where scCO_2_ acts as a co-solute and a pneumatic agent. In the SAA process, a controlled amount of scCO_2_ is first dissolved in a solution containing the components to be precipitated. The resulting expanded solution is then spray-dried into a precipitation chamber under atmospheric conditions, leading to the formation of fine particles [4]. Wu and coworkers (contribution 7) successfully complexed Beclomethasone dipropionate with γ-CD in the presence of leucine as a dispersion enhancer. The authors obtained spherical particles with excellent aerosol performance, and in vitro dissolution tests revealed a significantly faster release rate for Beclomethasone dipropionate, which completely dissolved within 60 min, compared to a 36 h dissolution time for the unprocessed drug.

## 3. Conclusions

The articles in this Special Issue highlight the growing potential of supercritical techniques in pharmaceutical manufacturing. Attention is focused on investigating how the operational parameters of the supercritical process influence not only the morphologies and physical states of drugs in formulations but also their dissolution rates and pharmacokinetics. Different areas are covered, including colonic delivery, tissue engineering, anticancer therapies, and pulmonary treatments. The contributions presented here will undoubtedly help the gradual shift of supercritical techniques from research to industrial applications, a shift we believe will occur in the near future.
